# Functional reprogramming of melittin by Pluronic^®^ F-127 enables anticancer selectivity with attenuated hemolytic activity

**DOI:** 10.3389/fonc.2026.1823622

**Published:** 2026-05-13

**Authors:** Grzegorz Król, Angelika Mańkowska, Paulina Paprocka, Małgorzata Sidewicz, Jakub Spałek, Sławomir Okła, Ewelina Piktel, Robert Bucki

**Affiliations:** 1 Institute of Medical Science, Collegium Medicum, Jan Kochanowski University of Kielce, Kielce, Poland; 2 Department of Medical Microbiology and Nanobiomedical Engineering, Medical University of Białystok, Białystok, Poland; 3 Holy-Cross Cancer Center, Kielce, Poland; 4 Independent Laboratory of Nanomedicine, Medical University of Białystok, Białystok, Poland

**Keywords:** A549 adenocarcinoma cells, anticancer therapy, HeLa cervical cancer cells, hemolytic activity, melittin, Pluronic® F-127

## Abstract

**Introduction:**

Conventional anticancer treatments have a number of limitations, including significant toxicity to healthy cells. Therefore, it is necessary to explore new therapeutic methods and strategies that utilize bioactive compounds with high selectivity for cancer cells while simultaneously limiting toxicity. Melittin is a membrane-active antimicrobial peptide known for its significant anticancer potential; nevertheless, its pronounced hemolytic activity greatly restricts its therapeutic use. In this work, strategy to functionally separate anticancer efficacy from hemolytic toxicity using Pluronic^®^ F-127 (PF127) is investigated.

**Methods:**

A549 human lung adenocarcinoma and HeLa cervical carcinoma cell lines were subjected to treatment with melittin (0.5–50 µg/mL), PF127 (1–5%), and their combinations. Cancer cell viability was assessed using the MTT assay, and cell apoptosis was evaluated using flow cytometry. Cell migration capabilities of treated cancer cells were determined using scratch assay. The hemolytic activity of melittin and the melittin/Pluronic^®^ F-127 combination, an indicator of toxicity to cell membranes, was assessed using hemolysis of human red blood cells (RBCs).

**Results:**

Our studies confirmed that melittin displays anticancer effects against A549 and HeLa cell lines in a dose-dependent manner and this effect is further augmented in the presence of PF127. Migration experiments revealed significant suppression of cancer cell motility after exposure to melittin/PF127. Furthermore, the use of the melittin/PF127 combination reduced the hemolytic activity of melittin in relation to RBCs.

**Conclusions:**

Co-treatment of cancer cells with melittin and PF127 enables selective anticancer effects while attenuating hemolytic activity. The findings indicate that the toxicity of membrane-active peptides and their anticancer effectiveness can be separated through formulation employment, underscoring PF127 as a promising element in the strategic development of safer melittin-based anticancer approaches.

## Introduction

Malignant tumors are one of the leading causes of death worldwide and one of the most serious challenges of modern medicine in terms of both diagnosis and treatment. The incidence is approximately 19.3 million diagnosed cases and 10.0 million deaths per year (1, 2). The basis of neoplastic transformation is a cascade of molecular changes leading to deregulation of proliferation, inhibition of apoptosis, acquisition of the ability to uncontrolled growth and metastasis (3). Treatment for most localized solid tumors involves surgery, radiotherapy, and chemotherapy, either in combination or separately. Radiotherapy is used in more than half of cancer patients, and although the method is highly effective, it has short- and long-term adverse effects. Chemotherapy also brings some challenges, such as drug resistance and significant tissue damage (4–7). Therefore, it is necessary to search for new methods and therapeutic strategies that include compounds with preferential activity toward cancer cells while maintaining limited toxicity.

One of the promising directions of research on cancer therapies is the use of natural bioactive compounds from the antimicrobial peptides group. Among them, some special consideration is given to the anticancer peptide melittin (GIGAVLKVLTTGLPALISWIKRKRQQ). Melittin (Mel) is a 26-amino acid peptide isolated from bee venom (honeybee *Apis mellifera* Linnaeus, 1761). While mostly recognized for its antibacterial activities, melittin has been also demonstrated to display antiviral and antifungal properties (8–11). Mechanistically, the broad-spectrum effect of melittin is possible due to its positive charge and its amphipathic properties as melittin causes disintegration of the surface membrane by forming pores leading to loss of membrane permeability, depolarization and its fragmentation (12).

Melittin has been also presented as a promising anticancer agent with proven efficacy against various cancer cell lines, including lung adenocarcinoma (13, 14) and cervical carcinoma (15), where it modulates key signaling pathways involved in cancer progression, including proliferation, apoptosis, metastasis, and angiogenesis (11). Other malignancies against which melittin was shown to be effective include melanoma, prostate, ovaries, lungs, stomach, breast, bladder, leukemia and hepatocellular carcinoma (12, 16–18). However, despite its effective anticancer activity, the use of melittin in various types of proposed therapies has encountered many challenges and limitations related to its cytotoxicity towards eukaryotic cells. Notably, its instability and rapid degradation in the blood significantly reduces the therapeutic effect. Non-specific cytotoxicity manifested by serious toxic effects such as hemolysis, necrosis or activation of pain receptors also hinders its clinical use. Therefore, improving the functional balance between anticancer activity and off-target toxicity remains a major challenge in the development of melittin-based strategies. In this manner, one of the most promising molecules is PF127– a FDA-approved a non-ionic triblock copolymer composed of PEO–PPO–PEO - poly(ethylene oxide)-poly(propylene)-poly(ethylene oxide)) segments (19) exhibiting amphiphilic and thermoreversible properties and the ability to self-organize into micellar colloidal structures. Due to its high biocompatibility and biodegradability, PF127 was demonstrated to be predisposed to applications in anticancer drug delivery systems. Preclinical study results indicate that PF127 may increase the therapeutic effectiveness of conjugated cytotoxic molecules by (i) inhibition of the activity of transporters associated with multidrug resistance (MDR), (ii) improved penetration of therapeutic molecules through cell membranes, and (iii) increased drug retention inside cancer cells (12, 20). At the same time, loading amphiphilic drugs into micelles consisting of Pluronics (particularly PF127) was demonstrated to considerably reduce the hemolytic effects of these compounds. For this reason, the aim of this study was to evaluate whether PF127 can modulate the biological activity of melittin *in vitro* and improve the functional balance between anticancer efficacy and hemolytic activity.

## Materials and methods

### Materials

Melittin used in this study was synthetized by Lipopharm.pl (Zblewo, Poland) and provided with a declared purity of ≥95%. The peptide was dissolved in PBS to prepare stock solutions and stored at 4 °C until use. Working solutions were freshly prepared prior to each experiment. Pluronic^®^ F-127 was purchased from Sigma-Aldrich (Saint Louis, Missouri, USA), suspended with shaking in PBS or cell culture medium to the final concentrations of 1, 2.5, and 5% and stored at 4 °C until use.

### Cell culture

A549 (CCL-185™) human lung carcinoma and HeLa (CCL-2™) human cervical adenocarcinoma cell lines were used in the study. Both cell lines were cultured in Dulbecco’s Modified Eagle’s Medium (DMEM) supplemented with 10% fetal bovine serum (FBS) and 1% of Antibiotic Antimycotic Solution, and maintained at 37 °C, with an atmosphere containing 5% CO_2_.

### Assessment of cell viability

Cell viability was determined using MTT assay, based on the ability of the enzyme mitochondrial dehydrogenase to convert the water-soluble tetrazolium salt (3-(4, 5-dimethylthiazol-2-yl)-2, 5-diphenyltetrazolium bromide) to insoluble formazan. To do so, A549 and HeLa were seeded at the density of 2.5x10^4^ and 1x10^4^ cells/wells, respectively and after 24-hour spreading were exposed to increasing concentrations of melittin (ranging from 0.5 to 50µg/mL), both alone and in the presence of 1%-5% of PF127. Upon 24-hour incubation, the precipitated formazan was dissolved in 100 µL of dimethyl sulphoxide (DMSO) to obtain a colored solution, the intensity of which was measured spectrophotometrically at 540 nm wavelength.

### Drug interaction analysis using the ZIP model

To distinguish between additive and synergistic effects of melittin and PF127, drug interaction analysis was performed using the SynergyFinder 3.0 platform. The analysis was based on cell viability data obtained from MTT assays following 24 h exposure to melittin (0.5–50 µg/mL) in combination with PF127 (1-5%). Melittin-PF127 interactions were evaluated using the Zero Interaction Potency (ZIP) model, which compares the observed response of drug combinations with the expected response under the assumption of non-interaction between the agents. Synergy scores were calculated across the full dose–response matrix for each cell line. For interpretation, synergy scores >10 were considered indicative of synergy, scores between −10 and 10 as additive or non-interactive, and scores <−10 as antagonistic.

### Assessment of cell apoptosis using flow cytometry

In order to evaluate the extend of cell death induced by melittin and the melittin/PF127 combination, a flow cytometry analysis was performed. After 24-hour incubation of cells with the indicated compounds, cells were harvested, washed twice with PBS buffer and stained using Guava^®^ Annexin Red Kit according to the manufacturer’s instructions prior analysis using Guava^®^ EasyCyte™ flow cytometer (Cytek Biosciences).

### Fluorescence microscopy of cytoskeletal organization

To assess treatment-induced morphological changes, fluorescence microscopy was performed following exposure of cells to melittin and PF127. After 24 h of incubation of cells with indicated doses of both agents, cells were washed with sterile PBS, fixed with 3.7% formaldehyde (15 min, room temperature), followed by twice washing with PBS and permeabilization for 5 minutes with ice-cold Triton X-100 (Sigma Aldrich, Saint Louis, Missouri, USA) at the final concentration of 0.1%. Next, actin cytoskeleton was labeled using a rhodamine phalloidin conjugate (ActinRed™ 555 ReadyProbes™ Reagent, ThermoFisher Scientific, 30min, room temperature) while nuclei were counterstained with a Hoechst 33342 (NucBlue™ Live ReadyProbes™ Reagent, ThermoFisher Scientific, 5min, room temperature) both purchased from ThermoFisher Scientific (Waltham, Massachusetts, USA).

### Scratch assay

Migration capabilities of melittin/PF127-treated A549 and HeLa cells were investigated using automated IncuCyte^®^ Live-Cell Analysis System (Essen BioScience). A549 and HeLa cells were seeded at the density of 2.5x10^4^ and 1x10^4^ cells/wells, respectively into 96-well Incucyte^®^ Imagelock Plates and cultured until full confluence was reached. Once confluent, uniform, linear scratches were made using the IncuCyte^®^ Wound Maker device (Essen BioScience). Upon washing of cells with PBS, melittin alone and melittin with PF127 were added directly to the wells. The plates were then placed in the IncuCyte chamber and time-lapse images of wounded cells were recorded for 48 hours. Post-assay, recorded images were processed by defining a scratch mask and a cell confluence mask using IncuCyte^®^ Cell Migration Analysis Software. The stages of wound closure were quantitatively assessed using the relative wound density (RWD) parameter, defined as the ratio of cell density in the damaged area to the cell density in the intact area, expressed as a percentage.

### Assessment of cell membrane toxicity using hemolysis test

The hemolytic activity of melittin in combination with PF127 was determined using human red blood cells (RBCs) upon 1-hour exposition. To do so, whole blood collected from the healthy donor was centrifuged (2500 rpm, 10min, 4°C), serum was collected and RBCs were washed with PBS twice. Such isolated RBCs were re-suspended in PBS (hematocrit ~5%) and then treated with melittin (0.1-50 µg/ml) for 1 and 24h at 37 °C. After incubation, samples were centrifuged at 2500 rpm for 10 minutes prior collection of supernatant and its transfer to new 96-well plate. The amount of hemoglobin released was measured spectrophotometrically at λ=540 nm using a Tecan Spark plate reader. The PBS and 1% Triton X-100-exposed samples were considered as negative and positive controls (0% and 100% hemolysis, respectively).

### Net functional selectivity index calculation

To assess the balance between anticancer efficacy and hemolytic toxicity, a net functional selectivity index was calculated for each condition using data obtained from MTT assay and hemolysis assay after 24 h incubation using the formula: Net functional selectivity index = cancer cell inhibition (%) – hemolysis (%). Positive values indicate conditions where anticancer activity exceeds hemolytic toxicity, whereas negative values indicate an unfavorable efficacy-to-toxicity profile.

### Statistical analysis

Results are presented as mean ± SD from performed repetitions. The significance of differences between tested samples and control was determined using the One-way ANOVA with Tukey multiple comparisons test, p<0.05 was considered to be statistically significant. Statistical analyses were performed using OriginLab software. Linear regression parameters, including slope and Pearson correlation coefficient (r), were calculated using OriginLab software.

### Ethical considerations

The studies involving collection of whole blood from healthy donors were approved by Bioethics Committee of the Collegium Medicum of the Jan Kochanowski University in Kielce (2/2022). The studies were conducted in accordance with the local legislation and institutional requirements. The participants provided their written informed consent to participate in this study.

## Results

### PF127 modulates the cellular response to melittin in cancer cells

**Figure 1:**
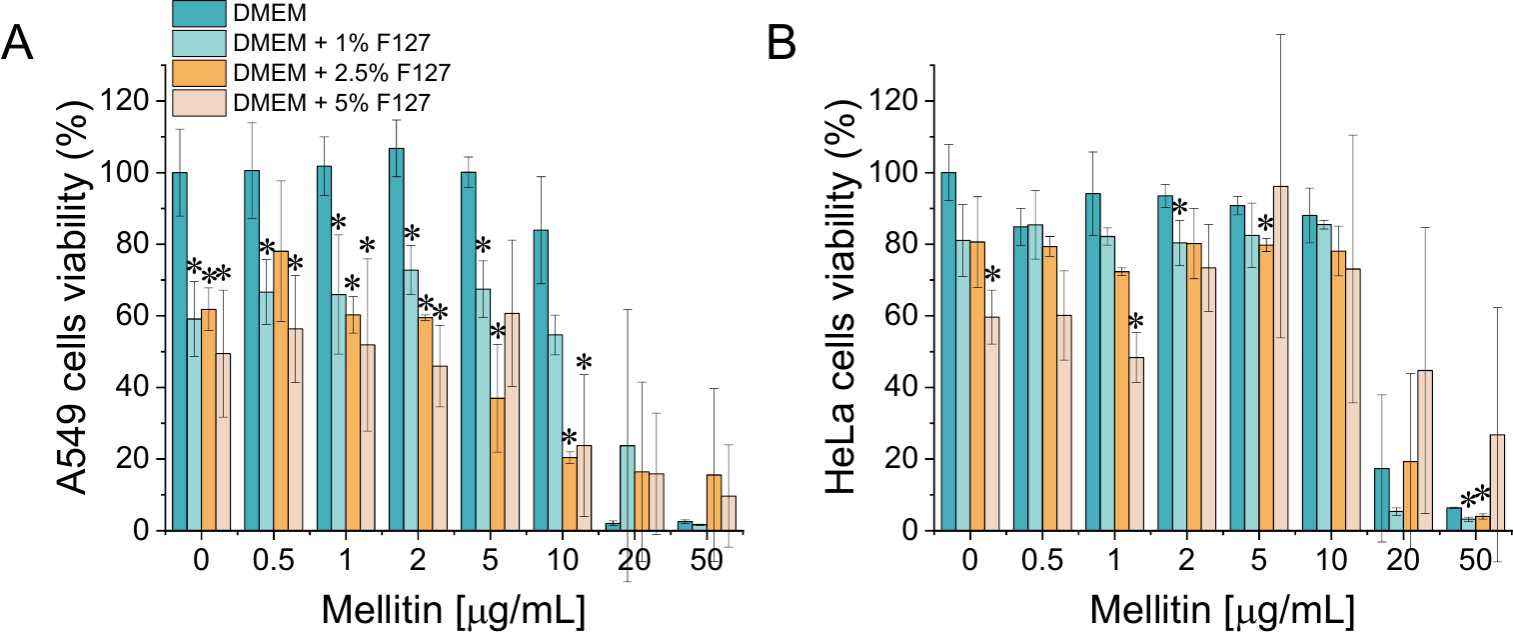
Cell viability of lung adenocarcinoma cell line A549. **(A)** and cervical cancer cell line Hela **(B)** upon treatment with melittin at doses of 0.5-50 µg/ml alone or melittin in combination with PF127 at final concentrations of 1%, 2.5%, and 5%. *denotes statistical significance compared to sample without PF127 addition; * p ≤ 0.05.

The cytotoxic effects of melittin, PF127, and their combinations were evaluated in A549 and HeLa cancer cell lines using the MTT assay. As shown in **Figures 1A, B**, in both tested cell lines, exposure to melittin resulted in a dose-dependent decrease in cell viability, with the strongest effect observed upon melittin addition at concentration of 20 or 50 µg/mL, consistent with strong cytotoxic properties of melittin. Significant effect was also observed for PF127 alone with A549 cells exhibiting greater sensitivity as application of 5% PF127 resulted in decrease of cellular metabolic activity up to 49.45% (**Figure 1A**). Co-treatment with melittin and PF127 resulted in further reduction in cell viability relative to melittin alone. The analysis of dose–response profiles indicated that the inclusion of PF127 modified the magnitude of the cellular response, resulting in reduced cell viability and demonstrating increased cytotoxic effects under combined treatment. Notably, this impact was noted in both cancer cell lines and was more pronounced at higher concentrations of PF127. Importantly, these results indicate that PF127 enhances the cytotoxic effect of melittin in cancer cells; however, this observation should be interpreted in the context of its concurrent effects on non-cancerous cells, as addressed in subsequent analyses.

**Figure 2:**
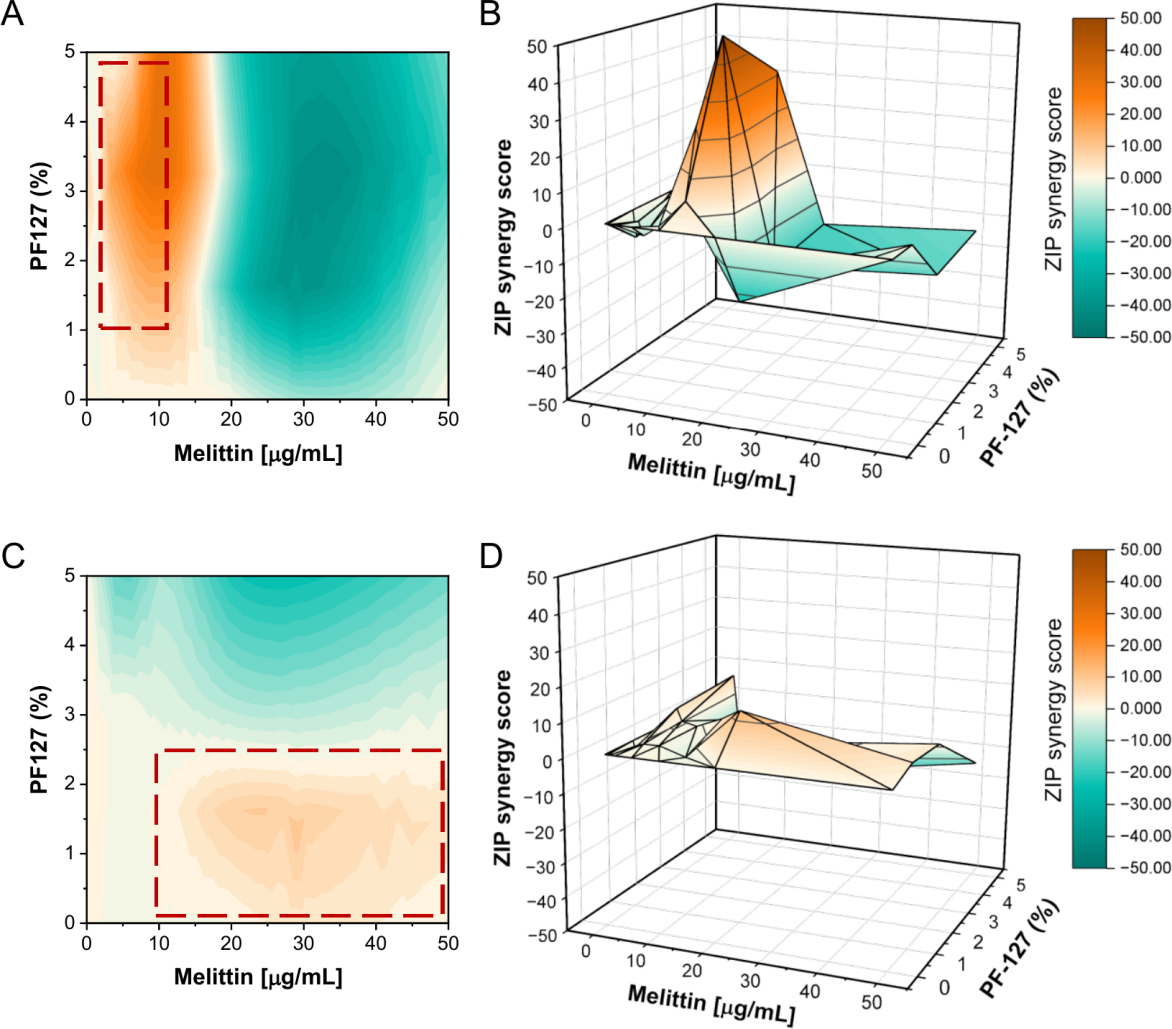
ZIP-based synergy analysis of melittin and PF127 interactions in A549. **(A, B)** and HeLa **(C, D)** cells. Drug interaction contour plots **(A, C)** and 3D color surface plots **(B, D)** were generated using the Zero Interaction Potency (ZIP) model based on cell viability data obtained after 24 h of treatment with melittin (0.5-50 µg/mL) in combination with PF127 (1-5%). Heatmaps represent synergy scores across the full concentration matrix, where values larger than 10 indicate synergistic interactions, values in a range of - 10 to + 10 indicate additive effects, and values < -10 indicate antagonism.

To distinguish between additive and non-additive interaction effects of melittin/PF127 co-treatment, a ZIP (Zero Interaction Potency) synergy analysis was performed for both tested cell lines (**Figure 2**). The analysis revealed that the interaction pattern was strongly dependent on both concentration and cell type. As demonstrated in **Figures 2A, B**, in A549 cells, a distinct synergistic region was observed at intermediate melittin concentrations combined with 2.5-5% PF127, with the highest ZIP scores noted for 10 µg/mL melittin + 2.5% PF127 (ZIP = 47.81), 10 µg/mL melittin + 5% PF127 (ZIP = 31.09), and 5 µg/mL melittin + 2.5% PF127 (ZIP = 20.80). These effects were localized and did not extend across the full concentration range. In contrast, combinations containing higher melittin concentrations (20–50 µg/mL) tended to show additive or antagonistic interactions. In HeLa cells (**Figures 2C, D**), the interaction pattern was less pronounced and was dominated mainly by additive effects, with only limited regions of synergy (ZIP > 10), observed for 10 µg/mL melittin + 1% PF127 (ZIP score = 12.2). Moreover, combinations containing 5% PF127 showed antagonistic interactions at several higher melittin concentrations. Overall, these data indicate that PF127 modulates melittin-induced cytotoxicity in a concentration- and cell line-dependent manner, rather than producing a uniform synergistic effect across the full concentration range.

### Melittin alone and when combined with PF127 induces apoptosis-associated cell death features in cancer cells

**Figure 3:**
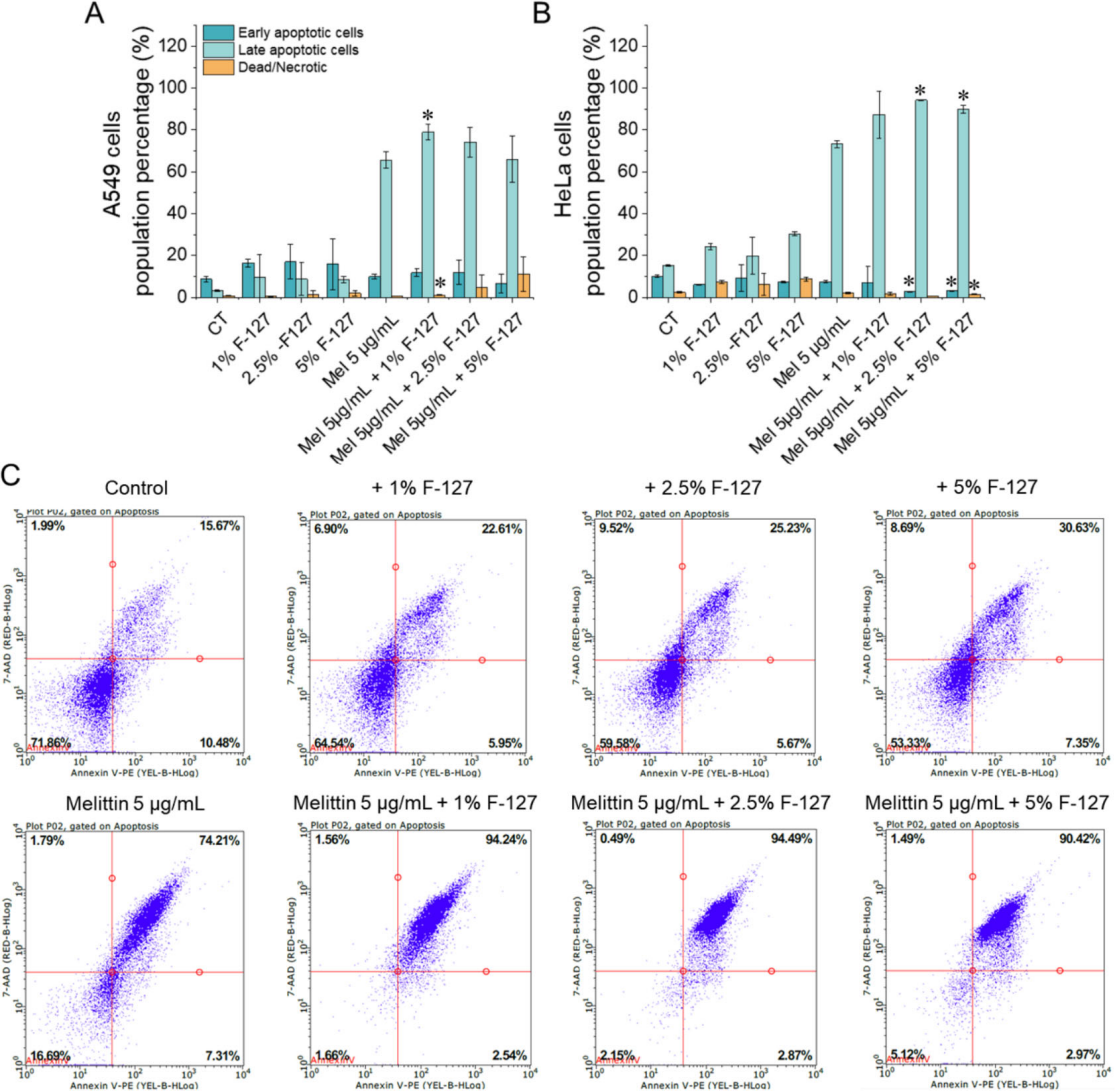
Annexin V-FITC/7-AAD flow cytometry analysis showing the percentage of apoptotic, and dead/necrotic A549 **(A)** and HeLa cells **(B)** after 24 h of treatment with melittin (5 µg/mL) and melittin combined with PF127 at final concentrations of 1%, 2.5%, and 5%. For the clarity of data presentation, population of live cells was not demonstrated. Representative flow cytometry plots for HeLa cells are represented in panel **(C)** *denotes statistical significance compared to sample without PF127 addition; *p ≤ 0.05.

To further elucidate whether the reduction in cancer cell metabolic activity observed in the MTT assay reflected apoptotic cell death features rather than exclusively nonspecific cytotoxic effects, flow cytometric analysis of apoptosis was performed using annexin V-FITC/7-AAD double staining. The employment of both annexin V and 7-AAD allowed the division of treated A549 and HeLa cells into four groups: (i) early apoptotic cells [annexin V(+)/7-AAD(–)], (ii) late apoptotic/dead cells [annexin V(+)/7-AAD(+)], (iii) dead cells [annexin V(–)/7-AAD(+)], and (iv) live cells [annexin V(–)/7-AAD(–)]. The calculated percentages of necrotic and apoptotic cells upon exposure to melittin and melittin/PF127 combinations are presented in **Figure 3**. In both cell lines Pluronic^®^ F-127 alone induced a moderate elevation in the percentage of Annexin V–positive cells, predominantly within the early apoptotic subpopulation. In contrast, melittin treatment at a concentration of 5 µg/mL led to a significant increase in the percentage of late apoptotic and dead cells indicating a pronounced cytotoxic effect of melittin against A549 and HeLa cells. Notably, the addition of PF127 potentiated this effect. Importantly, the percentage of necrotic cells remained low under the tested conditions. However, given the membrane-active nature of melittin, these results should be interpreted cautiously, as Annexin V/7-AAD staining does not allow definitive discrimination between primary apoptosis and secondary membrane disruption. Collectively, Annexin V-FITC/7-AAD dual-staining demonstrated that Pluronic^®^ F-127 modulates the pattern of melittin-induced cancer cell death, with an increased contribution of Annexin V–positive cell populations, rather than indicating a predominance of immediate necrotic cell death.

**Figure 4:**
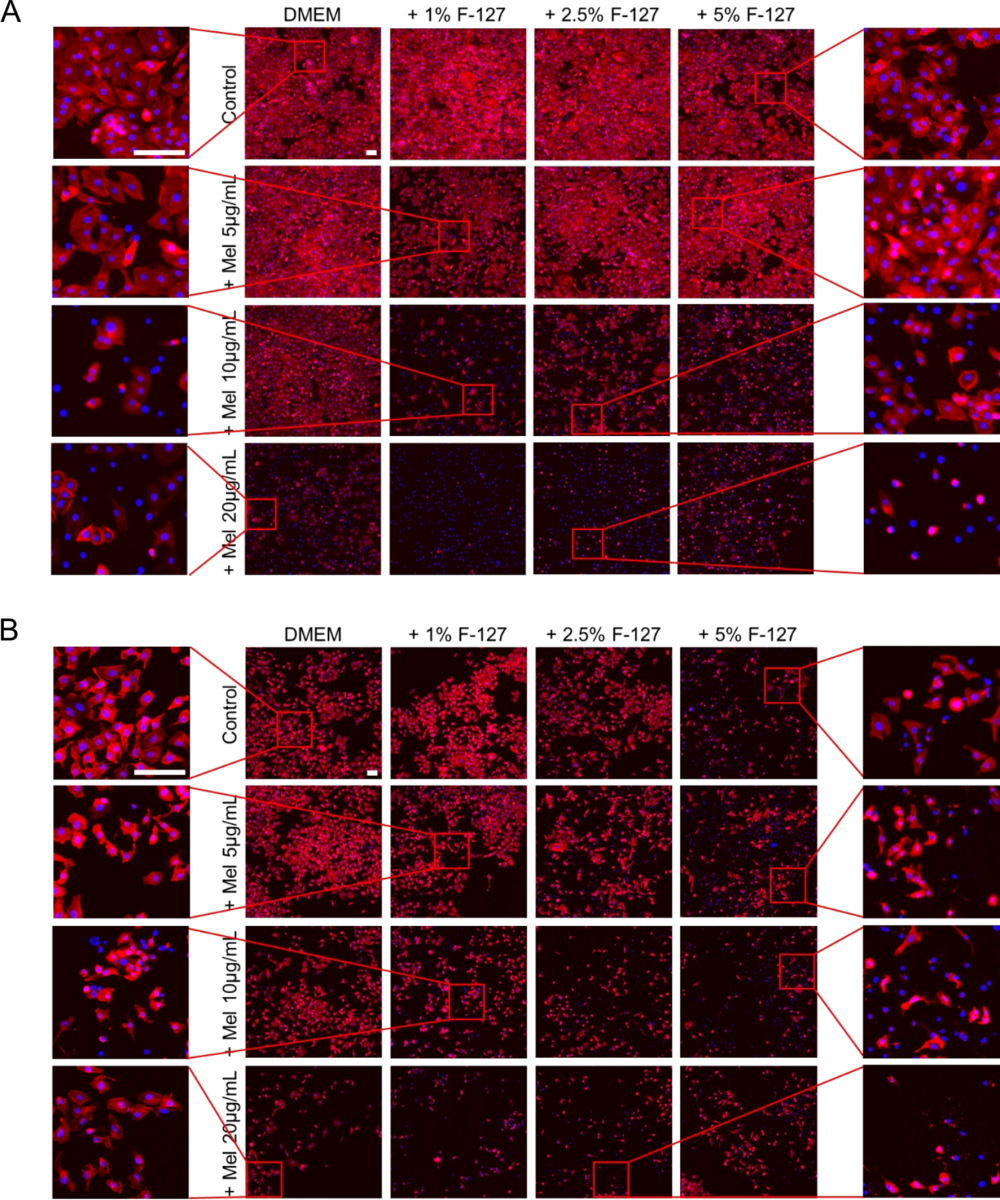
Representative fluorescence microscopy images of A549 **(A)** and HeLa **(B)** cells showing cytoskeletal organization (red) and nuclei (blue) in control and treated cells. Scale ~ 100µm.

In addition to flow cytometry analyses, fluorescence microscopy was employed to evaluate morphological changes in cells following treatment with melittin in the presence or absence of PF127. As demonstrated in **Figure 4**, control cells displayed typical morphology characterized by a well-spread cytoplasm and organized cytoskeletal structure. At the same time, treatment with melittin induced concentration-dependent changes, including reduced cell density, loss of cell spreading, and alterations in cytoskeletal organization. The presence of PF127 further modulated these effects and at higher PF127 concentrations, cells exhibited more pronounced morphological alterations, including increased rounding and reduced adherence, particularly in combination with melittin. Moreover, nuclear staining enabled visualization of nuclear distribution across conditions, and treated cells showed changes consistent with treatment-induced cellular stress. Overall, the observed morphological alterations are consistent with cell death–associated morphological changes, including features frequently observed during apoptosis, as well as reduced cell viability and support the cytotoxic effects identified in quantitative assays.

### Melittin/PF127 co-treatment significantly and dose-dependently inhibits the migration of the tested cells

**Figure 5:**
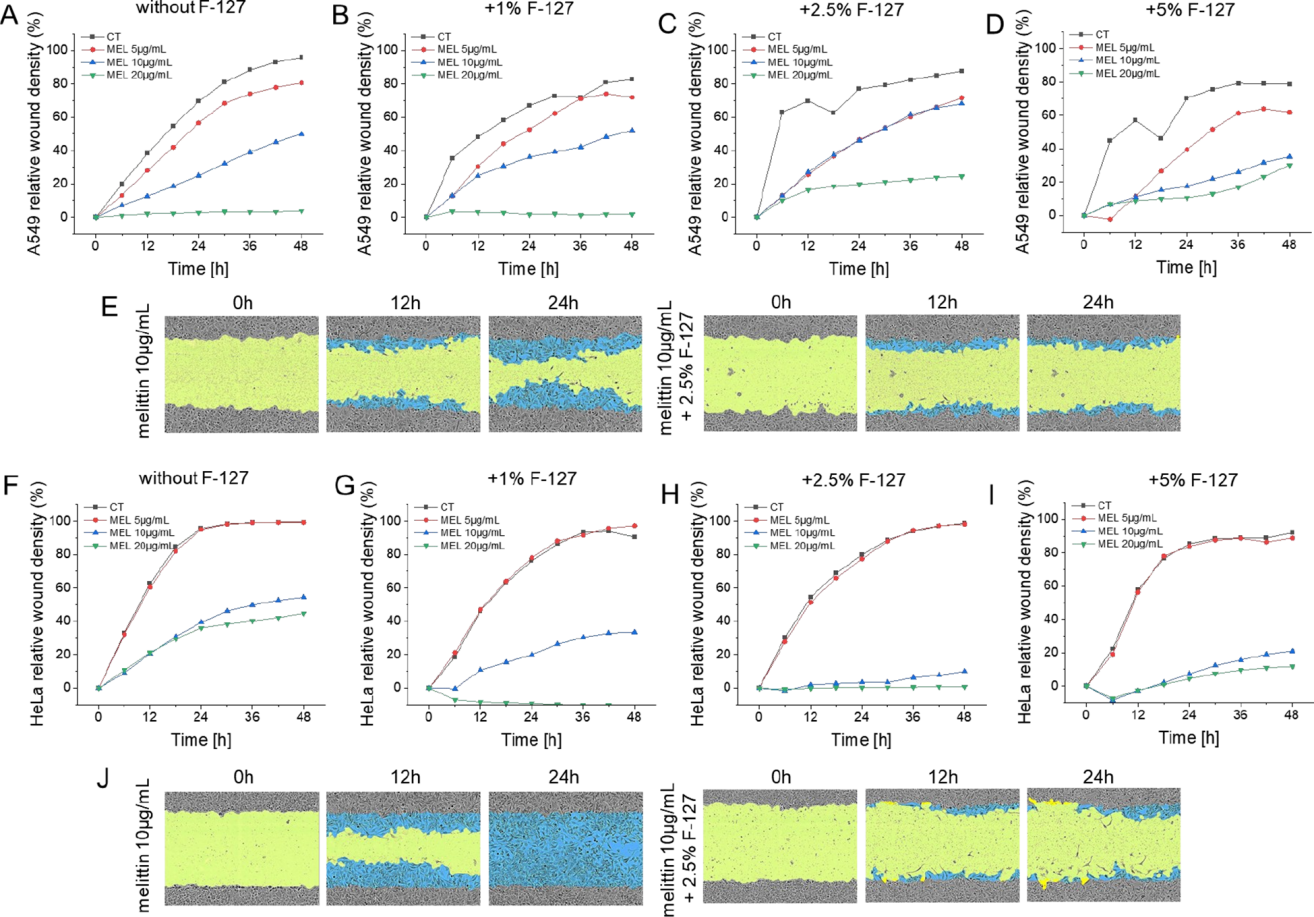
A549 **(A–E)** and HeLa **(F–J)** cancer cell migratory capabilities in the presence of melittin and PF127. Relative wound density of A549 and HeLa cells was calculated in the presence of melittin at doses of 5µg/mL (red lines), 10µg/mL (blue lines), and 20 µg/mL (green lines) both alone and in the presence of PF127 at the final concentrations of 1%, 2.5%, and 5%. Representative images of wound masks applied to wounds created on A549 and HeLa cells, are provided in the **(E, J)**.

A scratch assay was performed to determine whether melittin alone or in combination with PF127 will affect the migratory properties of the tested cancer cell lines. As demonstrated in **Figure 5**, in both A549 and HeLa control cells, wound closure was progressive indicating that both cell lines preserved migratory capabilities under experimental conditions. Treatment of cells with PF127 alone reduced migration of cells, but this was prominent only for A549 cells and in a dose-independently manner. In contrast, melittin treatment led to a considerable reduction of wound closure in both A549 and HeLa cells. Combined treatment, in comparison to melittin alone, further suppressed wound closure. In A549 cells, co-treatment led to minimal wound closure over a 48-hour period, particularly at higher melittin concentrations and an increase in PF127 content. Similar trends were observed in HeLa cells.

### PF127 modulates the relationship between hemolysis and anticancer activity

**Figure 6:**
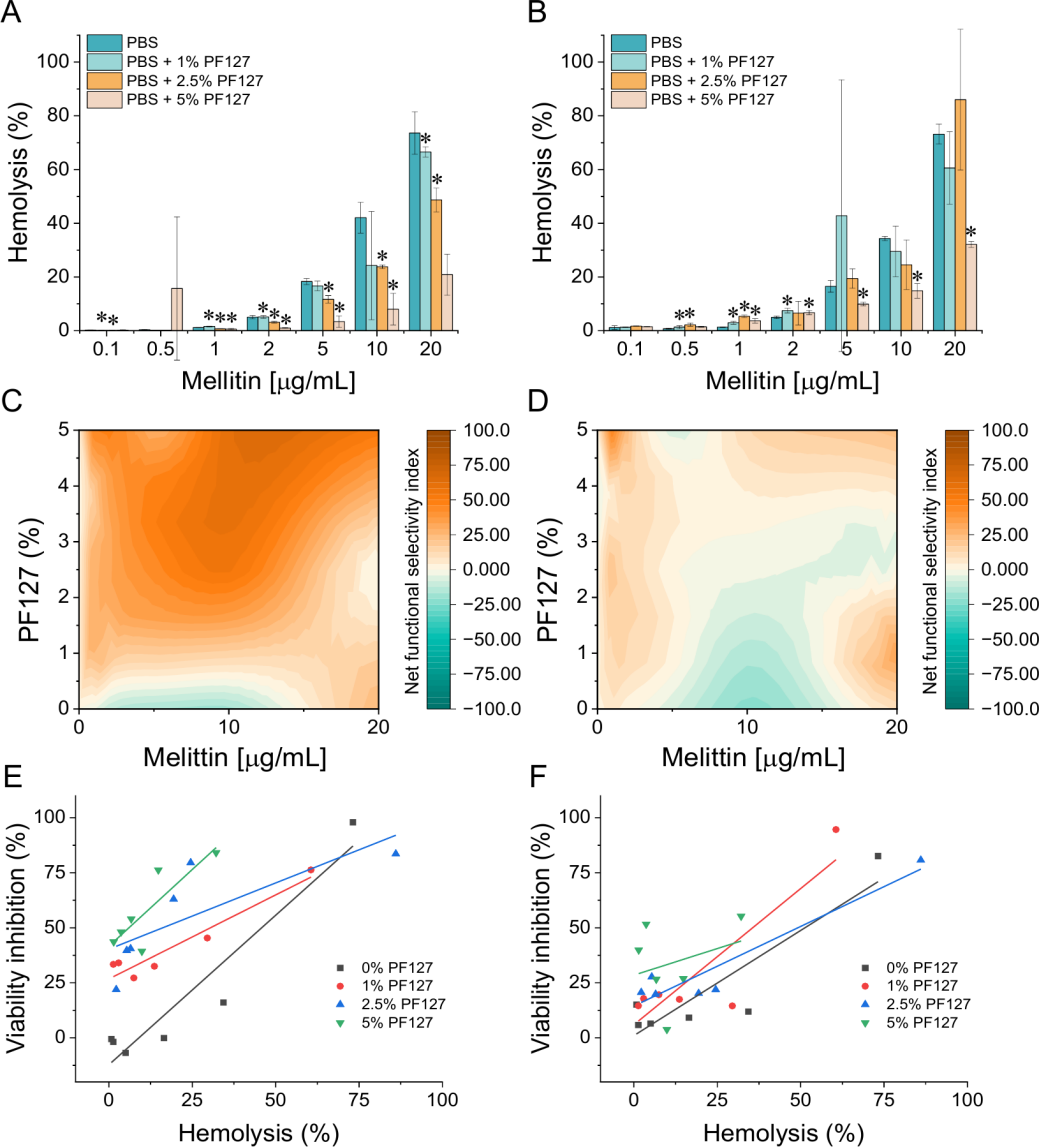
Time-dependent hemolysis, net functional selectivity, and correlation between hemolysis and anticancer activity in melittin/PF127-treated cells. Release of hemoglobin from human erythrocytes exposed for 1 **(A)** or 24 hours **(B)** to melittin at doses ranging from 0.1 to 50 µg/mL, both alone and in the presence of PF127 at the final concentrations of 1%, 2.5%, and 5%. *denotes statistical significance compared to sample without PF127 addition; *p ≤ 0.05. Interpolated landscapes of the net functional selectivity index for A549 **(C)** and HeLa **(D)** cells, calculated as cancer cell inhibition minus hemolysis measured after 24 hours. Positive values indicate conditions in which anticancer efficacy exceeded hemolytic toxicity, whereas negative values indicate an unfavorable efficacy-to-toxicity balance. Interpolated landscapes were generated using OriginPro based on discrete experimental data points across melittin concentrations and PF127 levels. Linear regression analysis of the relationship between hemolysis (%) and cancer cell inhibition (%) for A549 **(E)** and HeLa **(F)** cells under different PF127 concentrations.

The elucidation of PF127 potential to modulate the hemolytic activity of melittin was one of the main aims of this report. Indeed, it was noted that while melittin caused low hemolysis after 1 and 24 hours at doses ranging from 0.1 to 2 µg/mL, at higher doses, a significant increase in melittin-induced hemolysis was observed (**Figures 6A, B**). Notably, the use of PF127 reduced melittin-induced hemolysis under selected conditions. This effect was concentration-dependent, with more pronounced reductions generally observed at 2.5% and 5% PF127, particularly at melittin concentrations of 10 and 20 µg/mL. To further compare the efficacy and toxicity of proposed co-treatment, a net functional selectivity index was calculated. It was showed that PF127 improved the balance between anticancer activity and hemolysis in a concentration- and cell line-dependent manner, with a broader range of favorable conditions in A549 cells (**Figure 6C**) than in HeLa cells (**Figure 6D**). Moreover, linear regression analysis of hemolysis versus cancer cell inhibition demonstrated that, for melittin alone, anticancer activity was strongly associated with hemolysis in both cell lines. In contrast, PF127 modified this relationship depending on concentration. In A549 cells, the most favorable effect was observed at 2.5% PF127 (**Figure 6E**), whereas in HeLa cells the weakest association between hemolysis and anticancer activity was observed at 5% PF127 (**Figure 6F**). Collectively, these findings indicate that PF127 can partially improve the functional efficacy-to-toxicity balance of melittin, although this effect is strongly context-dependent.

## Discussion

Natural compounds such as melittin are currently highly recognizable in the development of novel cancer treatment methods. To date, a considerable number of data demonstrated high anticancer potential of melittin and melittin-derived peptides. Studies conducted using different cell line and animal models indicate the ability of these peptides to inhibit the proliferation of breast cancer, ovarian cancer, cervical cancer, prostate cancer, bladder cancer, hepatocellular carcinoma, brain cancer, colon cancer, melanoma and hepatocellular carcinoma cells (15, 21–28). Varied mechanisms of anticancer activities of melittin were demonstrated to date. In one of the studies, melittin was shown to effectively limit melanoma cell growth and migration by simultaneously inhibiting the phosphatidylinositol-3-kinase/protein kinase B/mTOR serine-threonine kinase (PI3K/AKT/mTOR) and mitogen-activated protein kinase (MAPK) pathways (16). Melittin was also demonstrated to cause calcium influx and induces apoptosis in osteosarcoma and stomach cancer cells (29, 30). Literature data also suggest potential positive applications of melittin in leukemia. It inhibits the growth of human promyelocytic leukemia granulocytes by inhibiting calmodulin and can induce cytolysis in human monocytic leukemia cells. It has also been shown to induce apoptosis in acute lymphoblastic leukemia and chronic myeloid leukemia cell lines (31, 32). All these reports clearly confirm that melittin displays significant efficacy by inducing apoptosis and necrosis, preventing angiogenesis and cell cycle arrest, and inhibiting cancer cell metastasis. However, its clinical use has limitations related to its toxicity, degradation, and inefficient systemic transport (12). Particularly, melittin is characterized by amphipathic features, which results in strong interactions with biological membranes. Its insertion into the bilayer drastically increases membrane permeability with a rapid cytoplasm leakage occurring within a few minutes after melittin addition (33). As a result, water, ions, and other particles freely pass through the membrane, leading to cell death and tissue damage. Sufficiently high concentrations of melittin have a toxic, systemic, and potentially fatal effect (34).

Although evidence shows strong potential of melittin in cancer treatment, its lack of biocompatibility requires the development of novel strategies to reprogram the biological actions of melittin and allow for its use as a potential anticancer therapeutics. The main direction of these strategies is to design and synthesize modified melittin with reduced toxicity while possessing pharmacological anticancer activity, as well as to develop stable, biocompatible drug delivery carriers (improved targeting and controlled release of the compound) (35). In the present study, we demonstrate using MTT assay, apoptosis assay and scratch assay that melittin in highly cytotoxic against lung and cervical cancer cells. These results are consistent with previous publications carried out, among others, on the Hela cell line (36, 37) or a human thyroid cancer cell line (38). Moreover, analysis of wound closure assay indicates a significant inhibitory effect of melittin in both tested cell lines. The observed dose-effect relationship, expressed by reduced wound closure with increasing melittin concentration, confirms literature reports describing its anticancer activity, among others, in the study of melittin in combination with erlotinib in the A549 lung cancer cell line (13). Similar results were also observed in studies on the inhibition of migration of B16F10, A375SM and SK-MEL-28 melanoma cells (B16F10 – a murine melanoma cell line, A375SM – a human melanoma cell line with high metastatic potential, and SK-MEL-28 – a human melanoma cell line derived from skin metastases) in a dose-dependent manner with melittin (16). Importantly, although scratch assays are unable to differentiate between migration and proliferation, the ongoing inhibition of wound closure observed under combined treatment conditions suggests that aggressive cellular behavior is being suppressed, rather than an isolated metabolic effect.

Most notably, conducted experiments allowed us to evaluate whether PF127 can modulate the relationship between melittin-induced hemolysis and its anticancer efficacy using a biocompatible poloxamer Pluronic^®^ F-127. The selected concentrations of PF127 (1-5%) were intended to span a low-to-moderate effects in our experimental settings and allowed us to assess both individual and combined effects of melittin and PF127 across a relevant experimental window. Importantly, this range also enabled us to evaluate the concentration-dependent modulatory effect of PF127 on melittin activity, which was central to the aim of this study. We demonstrate that co-treatment of cancer cells with melittin and PF127 significantly increase the anticancer effects while reducing the hemolytic properties of melittin. Nevertheless, our findings indicate that PF127 does not uniformly potentiate melittin activity, but rather modulates its biological effect in a concentration-dependent manner, with a clear synergistic window in A549 cells and predominantly additive or partly antagonistic interactions in HeLa cells. Reports to date demonstrate that poloxamers, including Pluronic^®^ F-127 demonstrate the ability to act as biological response modifiers, increasing drug transport across different barriers. Interestingly, PF127 inhibits P-glycoprotein (P-gp), a key factor in overcoming multidrug resistance (MDR) in cancer cells. In the context of our study, reports demonstrating the ability of Pluronics to interact with cellular membranes, influencing lipid organization and membrane fluidity, and ultimately affecting cellular responses, including mitochondrial respiration, transduction of apoptotic signals or gene expression (39). Indeed, in the present work, PF127 reduced cancer cell metabolic activity and induced some apoptotic responses without affecting the hemolysis under selected conditions, indicating differential effects on cancer and erythrocyte membranes. A statement on apoptosis-associated features of cell death of melittin/PF127 combination rather than nonspecific membrane lysis is also supported by Annexin V-FITC/7-AAD dual-staining analyses – as demonstrated, co-treatment of cancer cells shifted the cellular population to apoptotic rather than necrotic ones, although this observation does not exclude the contribution of membrane-disruptive mechanisms.

A study demonstrating the restriction of hemoglobin release from melittin/PF127-exposed erythrocytes indicates that using an appropriate pharmaceutical formula allows the full activity of the substance to be maintained while limiting its toxic effect on healthy cells. As shown, the hemolysis of melittin-treated RBCs reached up to ~75%, which is in accordance with data on strong hemolytic effect of melittin on erythrocytes (40), while in PF127-modified samples, hemolysis drops to ~20% at the same melittin concentration. This observation seems to be potentially relevant from a translational perspective, particularly when systemic administration is considered, although further *in vivo* validation is required. The reason of such biological selectivity might be supported by well-established differences in membrane composition between cancer cells and erythrocytes. Particularly, cancer cell membranes are characterized by increased exposure of anionic phospholipids such as phosphatidylserine and altered membrane potential in contrast to erythrocytes, which possess highly ordered, cholesterol-rich membranes optimized for mechanical stability rather than signalling cascades (41–43). Melittin, as a strongly cationic, amphipathic peptide, exhibits preferential interaction with membranes with increased negative charge promoting in cancer cells the more stable binding and deeper insertion of the peptide (44). On the other hand, some pluronics (e.g. P85) were noted to selectively alter the membranes of pathological cells leaving those with non-pathological phenotype unresponsive to its exposure (39). This might suggest that PF127 alters the context of melittin’s interaction with the membrane, limiting its lytic effect on the ordered cholesterol membranes of RBCs.

For our study few limitations should be recognized. One of it is the lack of evaluation of the melittin/PF127 system in non-cancerous adherent cell models, such as fibroblasts. Although hemolysis assays provide important information on membrane toxicity in the context of potential systemic administration, future studies should include additional normal cell models to more fully evaluate the selectivity and safety of the proposed system. Another limitation of the present study is that apoptosis was primarily evaluated using Annexin V/7-AAD flow cytometry, supported by microscopy-based morphological assessment. While these approaches provide robust quantitative and phenotypic evidence of apoptosis-related cell death, they do not allow for detailed characterization of the underlying molecular pathways. Therefore, the present findings should be interpreted as indicating apoptosis-associated cellular responses rather than a complete mechanistic characterization of apoptotic signaling. Moreover, we do not provided physicochemical characterization of the melittin/PF127 system, including parameters such as particle size, zeta potential, morphology, and encapsulation efficiency. Therefore, the observed effects should be interpreted as PF127-mediated modulation of melittin activity rather than evidence of a structurally defined delivery system.

Collectively, our work provides evidence that the hemolytic and anticancer properties of melittin can be partially uncoupled within specific concentration ranges via interaction with PF127. At the same time, we acknowledge that the highest concentration tested (5%) exerted measurable biological effects by itself and thus should be interpreted as a biologically active formulation rather than an inert carrier. Future research concentrating on membrane-level mechanisms and sophisticated biological models will be crucial for elucidating the principles underlying this functional reprogramming and to guide the rational design of safer membrane-active anticancer drugs.

## Data Availability

The raw data supporting the conclusions of this article will be made available by the authors, without undue reservation.
